# Performance Evaluation of Enhanced Oil Recovery by Host–Guest Interaction of β-Cyclodextrin Polymer/Hydrophobically Associative Polymer

**DOI:** 10.3390/molecules30010109

**Published:** 2024-12-30

**Authors:** Xi Li, Zhongbing Ye, Pingya Luo

**Affiliations:** 1 State Key Laboratory of Oil and Gas Reservoir Geology and Exploitation, Southwest Petroleum University, Chengdu 610500, China; yezb@swpu.edu.cn (Z.Y.); luopy@swpu.edu.cn (P.L.); 2 School of Materials and Environmental Engineering, Chengdu Technological University, Chengdu 610031, China

**Keywords:** hydrophobically associative polymer, β-cyclodextrin polymer, enhanced oil recovery, host–guest inclusion, polymer flooding

## Abstract

In this work, a hydrophobically associative polymer (HAP) was mixed with β-cyclodextrin and epichlorohydrin polycondensate (β-CDP) in an aqueous solution to enhance the intermolecular interaction through host–guest inclusion between hydrophobes and cyclodextrins. Results showed that the host–guest interaction improved the thickening ability and viscoelasticity of the HAP solution and maintained its shear thinning behavior. The host–guest inclusion system demonstrated special viscosity–temperature curves and variable activation energy. Enhanced oil recovery (EOR) performance tests showed that the oil increment produced by the host–guest inclusion system was 5.5% and 9.3% higher than that produced by the HAP and the partially hydrolyzed polyacrylamide solution, respectively. Compared with pure HAP, β-CDP/HAP has a better comprehensive performance and is more attractive for EOR in high-temperature reservoirs.

## 1. Introduction

With the increasing global energy demand and the depletion of reserves, enhanced oil recovery (EOR) has become increasingly important [1,2,3]. As a robust EOR technology, polymer flooding has been widely used. In the polymer flooding process, water-soluble polymers are dissolved in an aqueous solution, which can correct the water–oil mobility (*M*) by thickening and reducing permeability, thereby increasing oil recovery [4]. Given its low cost and good thickening ability, partially hydrolyzed polyacrylamide (HPAM) is the major polymer used for EOR [5,6]. However, the thickening ability of HPAM decreases sharply under severe conditions, such as high temperature or high salinity. At a high shear rate (*γ*), the viscosity of the HPAM solution also decreases rapidly because the skeleton is snipped by the applied stress on the polymer. HPAM with a high molecular weight (long skeleton) has a high thickening efficiency and is susceptible to mechanical degradation [7,8]. These deficiencies limit the application of HPAM in harsh reservoir environments. Therefore, in recent decades, many studies have been carried out to change the chemical structure of HPAM to compensate for its shortcomings.

Hydrophobically associative polymers (HAPs) are modified acrylamide copolymers that can be obtained by introducing a small amount of hydrophobes (less than 2 mol%) into a copolymer skeleton [9,10]. The intermolecular association of hydrophobes can form reversible 3D network structures that significantly increase solution viscosity. Temperature and salinity can enhance the hydrophobic association to a certain extent. Thus, the viscosity of the polymer solution can be further improved in a suitable reservoir environment. In addition, hydrophobic associations that are disassembled by a high shear rate can reform after shearing is eliminated, which confers HAPs with good resistance to mechanical degradation. Therefore, HAPs are superior to HPAM in terms of thickening ability and adaptability to harsh reservoir environments due to the formation of reversible hydrophobic associations. In other words, the introduction of intermolecular noncovalent interactions is a promising method for the development of efficient and robust EOR polymers.

β-Cyclodextrin (β-CD) is a cyclic oligomer that is composed of seven glucose units connected by 1,4-α-glycosidic bonds [11]. In view of the fact that the rim of β-CD is hydrophilic and the cavity is hydrophobic, the cavity can selectively incorporate hydrophobes of the appropriate size to form a host–guest inclusion compound via the steric effect, hydrophobicity, and various interactions involving van der Waals forces, dispersive forces, dipole–dipole interactions, electrostatic forces, and hydrogen bonding. In recent years, cyclodextrins and their polymers have attracted extensive interest because of their ability to modulate the rheological properties of HAP solutions. β-Cyclodextrin polymer (β-CDP) with a fairly high molecular weight and HAP can produce strong noncovalent interactions, i.e., host–guest interactions, which can further increase the viscosity of the HAP solution [12,13,14]. Weickenmeier et al. [15] reported a thickener based on host–guest interaction between 4-tert-butylaniline (guest) and β-CD (host) groups, both of which were grafted onto poly[(maleic anhydride)-alt-(isobutene)]. They observed a significant increase in solution viscosity by two to three orders of magnitude. Li et al. [16] established an enhanced polymer network based on the inclusion of octadecyl (C18) and β-CD grafted onto polyacrylic acid and modulated solution viscosity by modifying the type and density of hydrophobes.

In addition to the reported cyclodextrin polymers in which cyclodextrins were grafted as dangling groups, another water-soluble cyclodextrin polymer in which cyclodextrin is integrated into the polymer skeleton exists. This type of polymer has a high cyclodextrin content and can be easily synthesized by a one-pot method [17,18]. It is expected to form more noncovalent connections with HAPs in an aqueous solution, i.e., provide a higher solution viscosity.

Previous studies have shown that the supramolecular interaction between β-CDPs and HAPs can form stronger network structures in solutions. However, the cyclohexanol content in the polymers obtained by homopolymerization or graft polymerization is relatively low. Polymers obtained through cyclodextrin and epichlorohydrin condensation polymerization, on the other hand, can have a very high cyclohexanol content. This structure provides more binding sites and hydrophobic interactions, which may result in specific rheological properties that change under certain conditions. Moreover, experimental studies on the supramolecular polymer system formed by β-CDPs and HAPs in porous media have not been reported. It is unclear whether the stronger network structure persists in porous media or is destroyed by shear forces, and whether it impacts the adsorption performance of the supramolecular polymer system.

In this work, a water-soluble cyclodextrin polymer β-CDP was prepared by the polycondensation of β-CD and epichlorohydrin (EP). An efficient thickening flooding system (β-CDP/HAP) was developed by the host–guest interaction of the water-soluble β-CDP and an HAP that has been applied in the field. The rheological properties of the flooding system were evaluated, including temperature resistance, salt resistance, shear resistance, and viscoelasticity. In addition, core experiments were performed to evaluate the mobility control and displacement performance of β-CDP/HAP.

## 2. Results and Discussion

### 2.1. Solution Properties of HPAM, HAP, and β-CDP/HAP

#### 2.1.1. Shear Thinning Property

When polymer solutions are injected into porous media as displacement fluids, their viscosity and injectability are the primary considerations for mobility control in the reservoir. Therefore, the relationship between the apparent shear viscosity (*η*_s_) and the shear rate curve provides insights into the structure of polymer chains [2]. The shear thinning properties of the aqueous solutions of HPAM, HAP, and β-CDP/HAP were studied at a temperature of 25 °C and a shear rate of 0.01–1000 s^−1^.

**Figure 1:**
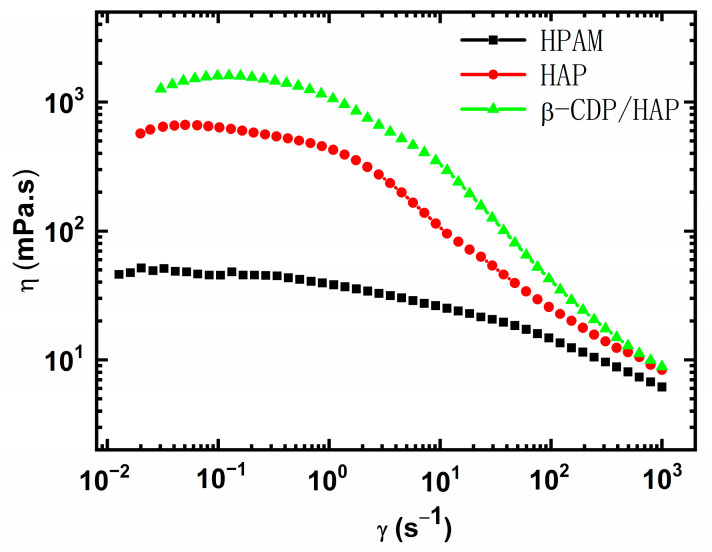
Apparent shear viscosity as a function of shear rate for the three polymer solutions in 4500 mg·L^−1^ NaCl brine (*C*_p_ = 1750 mg·L^−1^, *T* = 25 °C).

As shown in Figure 1, the relationship between the viscosity of the three polymer solutions and the shear rate can be divided into two parts: the Newtonian plateau or shear thickening region and the shear thinning region, where the apparent viscosity drops to the minimum. Previously, this phenomenon was repeatedly observed, and these experimental data are consistent with the improved Carreau model [19,20].

(1) $$\eta_{S}=\frac{\eta_{0}}{{\left(1+{\left(\lambda_{S}\times \gamma \right)}^{2}\right)}^{1-n/2}}$$

where *η*_0_ represents the zero-shear viscosity, mPa·s; *λ*_s_ is a time constant, with 1/*λ*_s_ being the critical shear rate (*γ*_c_), above which the viscosity begins to drop; and *n* represents the standard power law index.

Table 1 shows the parameters of the three polymer solutions, exhibiting the following distinctive features: (i) for all three polymer solutions, *n* is less than 1, showing the classic behavior of a power-law fluid; (ii) for pure HAP, *η*_0_ is much higher than that of the HPAM solution but lower than that of the β-CDP/HAP solution, indicating the presence of structural viscosity in the HAP and β-CDP/HAP solutions, while the structural viscosity is higher in the β-CDP/HAP solution than in the other solutions [9,21]; (iii) the ${\dot{\gamma }}_{c}$ value for HAP is smaller than that of HPAM, i.e., the transition between the zero-shear and power-law regions shifts to a lower shear rate for HAP and β-CDP/HAP. The difference at the onset of shear thinning supports the preferential disassembly of those aggregates formed by noncovalent bonds (hydrophobic association and host–guest inclusion), followed by the disentanglement of polymer chains [22]. In the low shear rate region, β-CDP/HAP exhibits the most obvious shear thickening phenomenon, which is often observed in HAP solutions [23,24,25]. The explanation for HAP solutions is that shearing leads to the transformation from intramolecular to intermolecular associations or causes the non-Gaussian chain extension of polymer chains [26].

For the β-CDP/HAP solution, the host–guest inclusion is dominant, and few hydrophobes can associate with each other. The shear-thickening phenomenon indicates that shear is conducive to the induced orientation and extension of polymer chains and further enhances the host–guest inclusion. Meanwhile, the most obvious shear thickening phenomenon implies that HAP chains exhibit a relatively coiled conformation due to inclusion interaction. With the increase in shearing rate, the reversible network structures formed by host–guest inclusion tend to dissociate, and the β-CDP/HAP and pure HAP solutions exhibit similar viscosities. The highest zero-shear viscosity is helpful for β-CDP/HAP to establish flow resistance in porous media, and the strongest shear thinning behavior gives it the highest injectability in reservoirs at a given concentration.

#### 2.1.2. Shear Resistance

When the shear stress exceeds the energy of the C-C bond of the polymer backbone, chain scission (i.e., mechanical degradation) occurs. This phenomenon occurs during polymer preparation, transportation, injection, and propagation in the reservoir [7,8]. It decreases the viscosity of the polymer solution, thereby reducing the polymer flooding effect.

**Figure 2:**
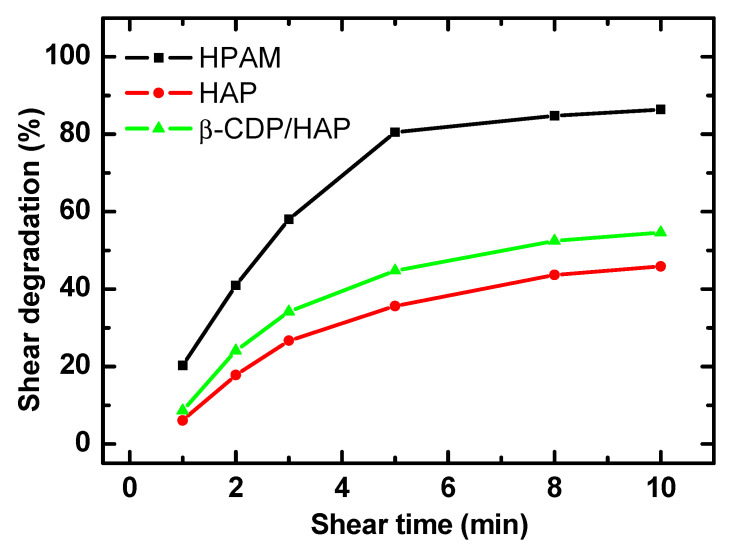
Shear degradation of polymer solutions (*C*_p_ = 1750 mg·L^−1^; Waring blender rotational speed, 6000 r/min).

Figure 2 plots the shear degradation of the polymer samples as a function of shear time. The shear degradation of the polymer samples initially increases sharply as the shear time is prolonged and then reaches a plateau. After shearing for 10 min, the viscosity loss of HPAM is about 86.4%, whereas that of pure HAP and β-CDP/HAP is approximately 45.9% and 54.6%, respectively. The shear degradation of HPAM is mainly caused by polymer chain scission. The hydrodynamic volume of HPAM decreases with shear time, which reduces the solution viscosity. Therefore, the viscosity of the solution decreases rapidly in the initial stage of shearing. However, since the molecular weight of HAP is smaller than that of HPAM, the mechanical degradation degree of polymer chains is lower, and new network structures are reformed through the hydrophobes or host–guest inclusion [13]. The shear degradation of the pure HAP and β-CDP/HAP solutions is lower than that of HPAM.

A comparison of the two polymer solutions with noncovalent bond association revealed that the shear degradation of β-CDP/HAP is slightly higher than that of pure HAP. This phenomenon may be related to the higher shear stress experienced by the β-CDP/HAP solution compared to pure HAP because of its higher viscosity. The β-CDP/HAP solution exhibits higher shear stress at the same shear rate, indicating that the additional inclusion in β-CDP and hydrophobes produces a stronger intermolecular interaction than that in hydrophobes. Even though the shear degradation of the β-CDP/HAP solution is higher than that of the HAP solution, it has the highest absolute apparent shear viscosity under the same conditions, due to its high initial viscosity.

#### 2.1.3. Salt Tolerance

**Figure 3:**
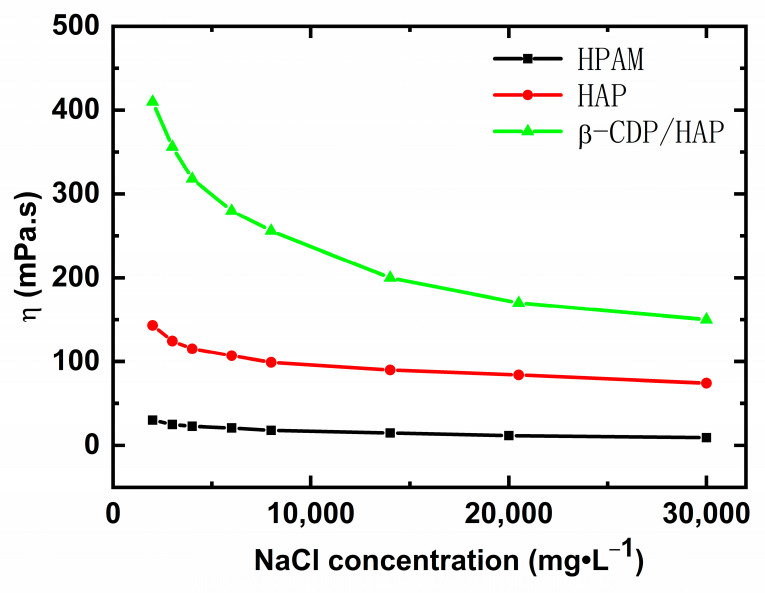
Effect of NaCl concentration on the viscosity of polymer solutions (*C*_p_ = 1750 mg·L^−1^; *γ* = 7.34 s^−1^).

The relationship between salinity and polymer solution viscosity is shown in Figure 3.

As the concentration of NaCl increases, the viscosity of the HPAM solution decreases and then reaches a plateau. This result can be ascribed to the fact that the electrostatic repulsion of the polymer chains decreases with increasing salinity, thereby reducing the hydrodynamic volume.

Although the increase in salinity promotes association and inclusion, the increasing electrostatic shield effect also reduces the thickening ability of pure HAP and β-CDP/HAP, resulting in a sharp decrease in the viscosity of the polymer solutions in the first stage. The same concentration of β-CDP/HAP solution has the highest viscosity in the low-salinity solution, whereas the viscosity retention of β-CDP/HAP (37%) is lower than that of pure HAP (52%) in the high-salinity solution (30,000 mg/L NaCl solution). This result indicates that salinity promotes the inclusion of hydrophobes with cyclodextrins, and the extremely coiled conformation of β-CDP causes HAP to present a coiled conformation [17]. The β-CDP/HAP solution has the highest retention viscosity under the same salinity condition. In addition, the host–guest inclusion between cyclodextrins and hydrophobes strengthens the association between HAP chains.

#### 2.1.4. Temperature Resistance

**Figure 4:**
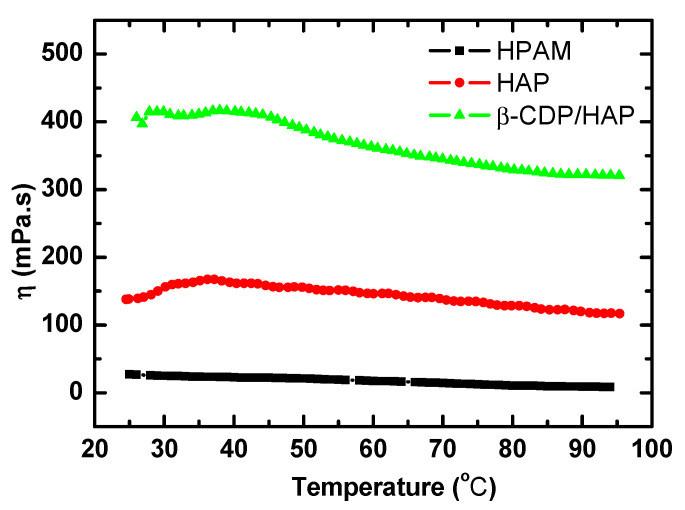
Effect of temperature on viscosity of polymer solutions (*C*_p_ = 1750 mg·L^−1^; *γ* = 7.34 s^−1^).

The relationship between polymer solution viscosity and temperature is shown in Figure 4.

The viscosity of the HPAM solution decreases unidirectionally with the increase in temperature, which is caused by the thermally induced movement of molecules. However, the viscosity of the β-CDP/HAP and pure HAP solutions initially increases to a peak and then decreases. Similar phenomena are consistently observed in other HAP solutions [2]. Although the two solutions present different solution viscosities at an identical temperature, the viscosity peaks for the two solutions appear at 35 °C. Therefore, the viscosity increase in this region should be ascribed to the thermal-enhanced hydrophobic association. As the temperature continues to increase, the intensified thermally induced movement of molecules weakens the aggregated structure formed by the hydrophobes, thereby weakening the intermolecular association between the polymer chains. As a result, the viscosity of the polymer solutions decreases.

To determine the influence of temperature on shear viscosity and the network structure formation mechanism of the three polymer solutions, we analyzed the temperature dependence of apparent viscosity by using the Arrhenius equation in the logarithmic form as follows:

(2) $$\ln\eta =\frac{E_{a}}{RT}+\ln A$$

where *A* is the pre-exponential factor, *E*_a_ is the free activation energy for viscous flow, *η* is the shear viscosity at the absolute temperature *T*, and *R* is the gas constant.

The HPAM and HAP solutions show a good linear relationship between ln*η* and 1/T in the temperature range of 35–95 °C, which is consistent with the flow kinetic theory represented by the above equation. The linear relationship between ln*η* and 1/*T* shows that the flow activation energy is constant and independent of temperature within the temperature range considered. For the HPAM solution, the flow activation energy calculated by the Arrhenius equation is 16.5 kJ/mol. Meanwhile, the calculated flow activation energy for the HAP solution is only 5.8 kJ/mol, which is lower than that of the HPAM solution and the results from other reports [27,28]. The magnitude of the flow activation energy, described as an energy barrier, usually represents the strength of interaction between polymer chains, and the HAP chains obviously have stronger interactions than the HPAM chains. However, the flow activation energy for HAP is lower because the flow activation energy calculated by the Arrhenius equation with the viscosity–temperature relationship only represents the temperature sensitivity of the polymer solutions. This unconventional result usually appears in temperature-thickening HAPs or thermoviscosifying polymers because a higher temperature promotes hydrophobic association and reduces the temperature sensitivity of the solution [22,29].

The Arrhenius equation between viscosity and temperature shows a progressive linear deviation in the β-CDP/HAP solution, which means that the flow activation energy varies with temperature. This result reflects the different connection mechanisms of the β-CDP/HAP solution network structures at different temperatures [28]. With the increase in temperature, the thermal motion increases, and the dissociation rate of the inclusion compound gradually increases. The number of hydrophobes that are not encapsulated by β-CDP increases, and these released hydrophobes form a new hydrophobic association in the aqueous solution. Therefore, the proportion of network structures formed by the host–guest inclusion and hydrophobic association differs at different temperatures, resulting in the activation energy varying with temperature. The variable activation energy indicates that this host–guest inclusion system can resist the adverse effects caused by the intensified thermal motion. As shown in Figure 4, the viscosity–temperature relationship curve of the β-CDP/HAP solution becomes almost a horizontal line above 85 °C. Temperature insensitivity helps this type of host–guest inclusion system maintain higher viscosity in high-temperature reservoirs.

#### 2.1.5. Viscoelasticity

The viscoelasticity of polymers plays an important role in EOR. The traditional view is that polymer flooding exerts no obvious effect on the microscopic sweeping efficiency. However, an increasing number of studies have shown that polymers exert tensile effects on residual oil trapped in “dead” ends, and improved viscoelasticity increases the microscopic sweeping efficiency [30,31].

**Figure 5:**
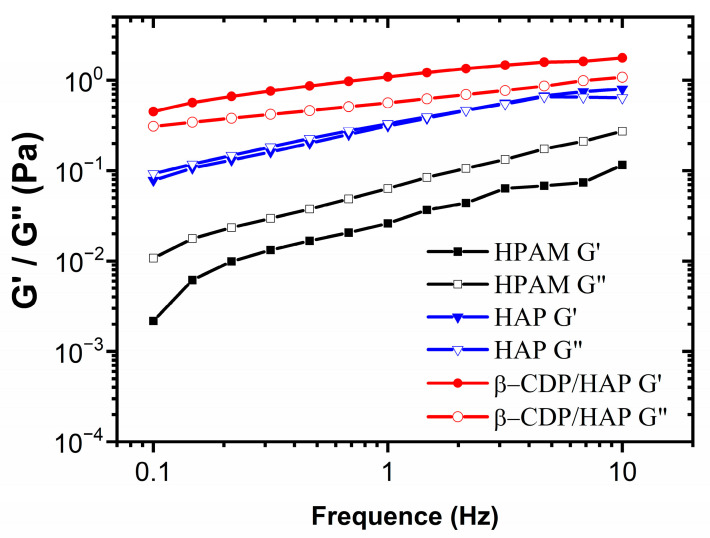
The frequency sweep curve for the polymer solutions (*C*_p_ = 1750 mg·L^−1^; *T* = 45 °C).

As shown in Figure 5, the elastic modulus (*G*′) and viscous modulus (*G*″) show an upward trend as the frequency increases. The elastic modulus (G′) and viscous modulus (*G*″) of the pure HAP solution are significantly higher than those of HPAM. This result can be attributed to the connections formed by intermolecular hydrophobic associations. The viscous modulus and elastic modulus of β-CDP/HAP are significantly higher than those of pure HAP. In addition, the elastic modulus is greater than the viscous modulus throughout the test region, showing typical viscoelastic fluid characteristics. This phenomenon can be attributed to the stronger network connections provided by the host–guest inclusion. In the β-CDP/HAP solution, the hydrophobes of HAP are firmly encapsulated in the cyclodextrin hydrophobic cavity of β-CDP because of the interaction between steric and hydrophobic effects, forming strong network connections. Additional host–guest inclusion improves the viscoelasticity of the HAP solution.

### 2.2. Core Flooding Results

**Figure 6:**
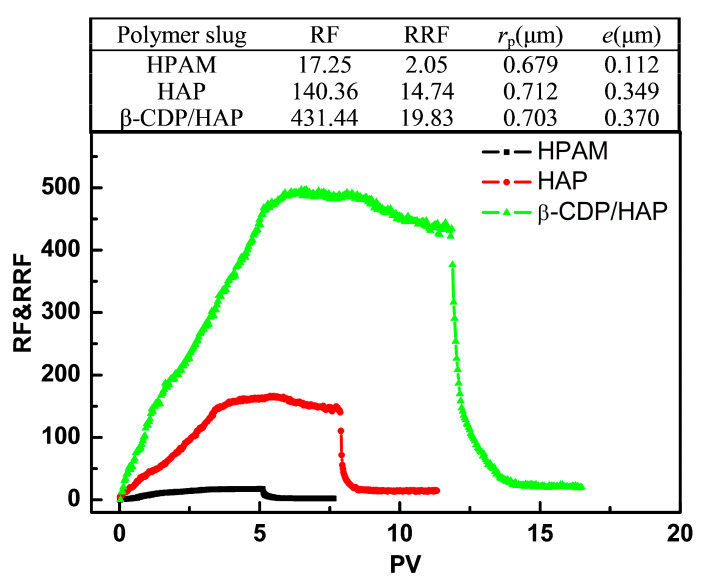
Resistance factor (RF) and residual resistance factor (RRF) as a function of pore volume (PV) of the polymer slugs (*C*_p_ = 1750 mg·L^−1^; ${\dot{\gamma }}_{w}$ = 7.34 s^−1^; *T* = 45 °C).

Figure 6 shows the flow behavior of the β-CDP/HAP, pure HAP, and HPAM solutions in porous media. The resistance factor (RF) is a measure of the ability to control mobility and provides information about the effective viscosity of polymer solutions in porous media. For the three polymer solutions, the RF value rapidly increases and gradually stabilizes during the injection of the polymer. The highest RF value of β-CDP/HAP means that it exhibits the highest effective viscosity and mobility control among all solutions. This phenomenon is a key factor for successful polymer flooding [8,32].

The highest RF value for β-CDP/HAP is attributed not only to its highest shear viscosity but also to its shear recovery ability. Strong intermolecular interactions induced by host–guest inclusion increase resistance to extensional flow [33]. In general, the flow behavior of the β-CDP/HAP solution through porous media is consistent with its rheological behavior in the low shear rate region.

The residual resistance factor (RRF) is used to evaluate the permanent permeability reduction in a porous medium due to polymer retention. Permeability reduction is mainly caused by polymer adsorption; the average hydrodynamic thickness of the polymer adsorption layer can be calculated using the RRF according to the Poiseuille equation [34,35]:

(3) $$e=r_{p}\cdot \left(1-RRF^{\left(-\frac{1}{4}\right)}\right)$$

where *e* is the average hydrodynamic thickness of the polymer layer (μm).

As shown in Figure 6, the RRF values of β-CDP/HAP and pure HAP are much higher than that of HPAM. HAPs generally show a larger RRF than conventional HPAM, which is consistent with previous reports. This result is usually attributed to the multilayer adsorption formed by the association of hydrophobes. The inner polymer is connected to the mineral surface by hydrogen bonds, whereas the loose outer layers are connected to the inner layer by hydrophobic interaction [34,35]. The multilayer adsorption of HAP can significantly reduce the permeability of porous media. The mechanism of the high RRF for β-CDP/HAP should also be similar. The host–guest inclusion allows the outer chains connected to the inner layer to form multilayer adsorption.

Comparison of the adsorption layer thickness values shows that HPAM forms a monolayer with a thickness comparable to its hydrodynamic radius in a dilute solution [22], whereas pure HAP and β-CDP/HAP form multiple thicker adsorption layers connected by intermolecular noncovalent bonds. The thickness of the adsorption layer of β-CDP/HAP is slightly higher than that of pure HAP, which may be due to the fact that the hydraulic radius of the aggregates formed by inclusion is larger than that formed by hydrophobic association. The remarkable permeability reduction ability gives β-CDP/HAP potential for the profile control of stratified or fractured reservoirs.

### 2.3. Enhanced Oil Recovery Experiment

**Figure 7:**
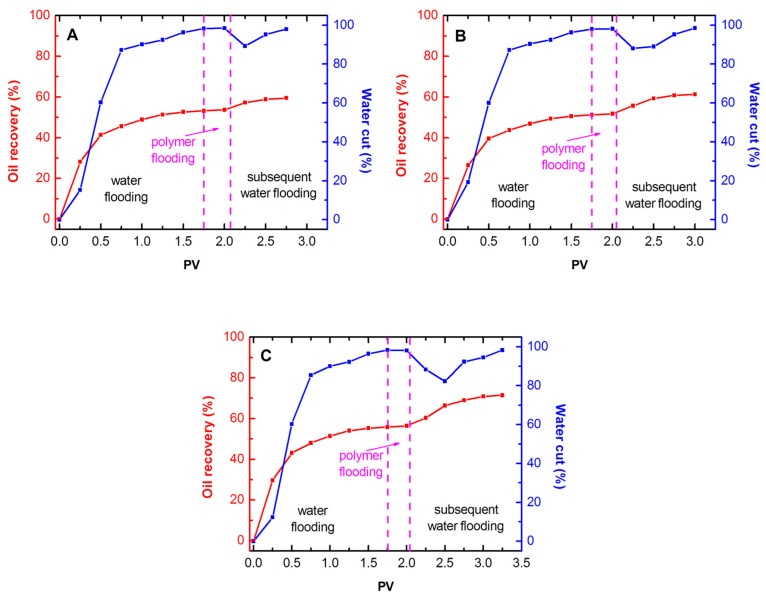
Cumulative oil recovery and water cut reported as a function of cumulative PV for (**A**) HPAM, (**B**) HAP, and (**C**) β-CDP/HAP (*C*_p_ = 1750 mg·L^−1^; *T* = 45 °C).

Figure 7 shows the details of polymer flooding. With the onset of water flooding, cumulative oil recovery continues to increase. The increase in cumulative oil recovery becomes very slow in the later stage because of the large viscosity difference between the crude oil and injection water. After about 1.75 PV water injection, the effluent water cut is close to 100%, and the cumulative oil recovery does not exceed 55.8% of the original oil in place. Then, a 0.3 PV polymer slug is injected, followed by subsequent water flooding. The cumulative oil recovery increases to different degrees.

The flow pattern of the polymer slug through a porous medium can be reflected by the distribution of cumulative oil recovery and the minimum water cut in the subsequent water flooding stage to a certain extent. Although the overall trends of oil increase in the subsequent water flooding are similar, the lowest oil production increment (6.3%) is obtained in the HPAM case. In the HAP case, the oil production increment is 10.1%. Meanwhile, the β-CDP/HAP slug flooding achieves the highest oil production increment (15.6%), showing the best EOR performance. In subsequent water flooding, the minimum water cuts of HPAM, β-CDP/HAP, and pure HAP are 89.3%, 88.1%, and 82.3%, respectively. The lowest water cut in the β-CDP/HAP case shows that the polymer slug significantly reduces the water phase flow capacity when flowing through porous media. This fact indicates that β-CDP/HAP may exhibit a “piston-like” propagation. Due to its strong intermolecular interaction, the viscosity and viscoelasticity loss are reduced in the continuous shearing and stretching process through the pore throat/pore.

The main reasons for the polymer flooding test results are as follows. The mobility of water and polymer injection can be expressed as follows:

(4) $$M_{w}=\frac{\mu_{o}K_{w}}{\mu_{w}K_{o}}$$

(5) $$M_{p}=\frac{\mu_{o}K_{p}}{\mu_{p}K_{o}}$$

where *μ* is the Darcy viscosity, and the subscripts w, p, and o are water, polymer, and oil, respectively. The ratio of *M*_w_ and *M*_p_ is the RF. In the HPAM case, the polymer’s effective viscosity is dominant in mobility reduction because of its monolayer adsorption. For HAP and β-CDP/HAP, the mobility reduction depends not only on the high effective viscosity but also on the reversible multi-layer polymer retention. Polymer flooding is not assumed to lower the residual oil saturation (S_or_). However, the improved sweep efficiency accelerates oil production, which can be understood by fractional flow curves [36]. The oil displacement becomes more effective as *M* decreases. In addition, the recovery increases nearly linearly with the logarithm of *M*. The trend does not depend on whether or not *M* is altered by permeability reduction or by viscosity increase. In the β-CDP/HAP case, the significant mobility reduction achieved by multi-layer adsorption helps reduce S_or_, which is a main goal for EOR. Combined with its highest viscosity and viscoelasticity, the maximum amount of crude oil can be recovered.

## 3. Materials and Methods

### 3.1. Material

**Figure 8:**
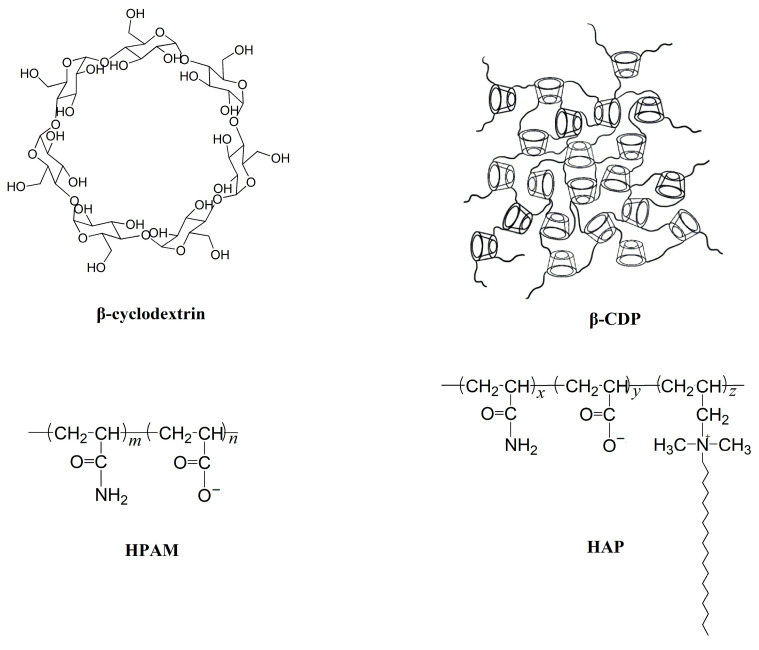
Schematic diagram of β-cyclodextrin, β-CDP, HPAM, and HAP.

The chemical β-cyclodextrin (β-CD) was purchased from Aladdin Biochemical Technology Co., Ltd., (Shanghai, China). Epichlorohydrin, sodium hydroxide (NaOH), acetone, and NaCl were all of analytical reagent grade and, along with hydrochloric acid (HCl), were all purchased from Chron Chemicals Co., Ltd., (Chengdu, China). HPAM (degree of hydrolysis = 25%; *M*_w_ = 1.6 × 10^7^; intrinsic viscosity = 19.4 dLg^−1^) and HAP (degree of hydrolysis = 21%; *M*_w_ = 6.6 × 10^6^; intrinsic viscosity = 21.7 dLg^−1^) were provided by Guangya Polymer Chemical Co., Ltd., (Chengdu, China). All chemicals were used without further purification. Deionized water was deionized twice with a Millipore Milli-Q system. Crude oil was obtained from the Dagang Oil Field (Tianjin, China). The apparent viscosity of the crude oil was 21.2 mPa·s at 45 °C and its density was 0.89 g·cm^−3^. Figure 8 shows a schematic diagram of β-cyclodextrin, β-CDP, HPAM, and HAP.

### 3.2. Synthesis of β-CDP

**Figure 9:**
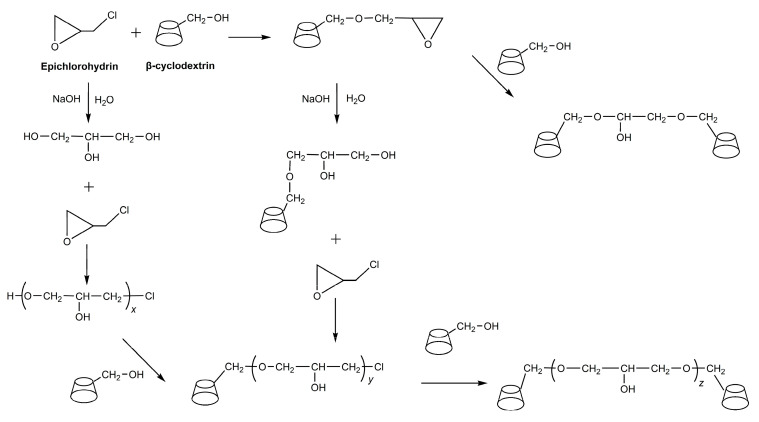
Schematic diagram of β-CD and epichlorohydrin condensation.

β-CDP was synthesized by the polycondensation of cyclodextrin and epichlorohydrin in an alkaline medium. Under alkaline conditions, the activated hydroxyl groups on β-cyclodextrin can react with epichlorohydrin to obtain products with side chains. The product can be further reacted to extend the side chain or polycondensate with other products to obtain cyclodextrins bridged by poly (2-hydroxypropyl) ether, which is shown in Figure 9. Details of its synthesis and structural characterization are described elsewhere [37,38]. A mixture of 5 g of β-CD (0.44 mmol) in 10 mL of NaOH solution (30 wt%) was mechanically stirred overnight at room temperature. The mixture was heated to 35 °C, and EP (4.709 mL) was quickly added. The reaction temperature was maintained at 35 °C. The reaction was stopped by the addition of acetone before colloid formation. The upper organic layer was removed by decantation. The pH of the aqueous solution was decreased to 12 by adding 1 mol/L HCl. Then, the solution was stored at 50 °C to hydrolyze overnight. After cooling, the solution was neutralized with HCl and diafiltrated (molecular weight cut-off of 20,000). Finally, the product was separated by freeze-drying. Under alkaline conditions, the activated hydroxyl groups on β-CD can react with epichlorohydrin to obtain products with side chains. The product can be further reacted to extend the side chain or polycondensate with other products to obtain cyclodextrins bridged by poly (2-hydroxypropyl) ether. The NMR spectrum of β-CDP in Figure S1 of the Supporting Information proves that CDP was successfully synthesized. Figure S2 provides information on molecular weight changes of products during the synthesis process. In order to study the thermodynamic stability of CD and CDP, analysis experiments were conducted, and the information is shown in Figures S3 and S4.

### 3.3. Evaluation of Solution Characteristics

The polymer-concentrated solution was obtained by uniformly dissolving the pre-weighed polymer at the required weight percentage (usually 0.5 wt%) in deionized water and leaving it still for 24 h. The polymer solution of the target concentration was obtained by dilution. After optimizing the component ratio, the host–guest inclusion polymer solution with the best rheological properties was obtained and denoted as “β-CDP/HAP” (HAP:β-CDP = 0.2:0.5 wt%).

As one of the most widely used polymers in polymer flooding, HPAM was employed for comparison. The solution salt and temperature resistance of HPAM, pure HAP, and β-CD/HAP were evaluated using viscosity measurements. All apparent viscosities were measured with a Brookfield DV-III viscometer at 7.34 s^−1^. The shear degradation of the polymer samples was evaluated using a Waring blender. Shear degradation can be quantified by viscosity loss (percentage), which is given in Equation (6):

(6) $$Shear\;degration=\frac{\eta_{b}-\eta_{a}}{\eta_{b}}\times 100\%$$

where *η*_b_ is the polymer solution viscosity before shearing, and *η*_a_ is the polymer solution viscosity after shearing.

The rheological properties of the polymer solutions were determined using an Anton Paar mcr302 rheometer. Small-amplitude oscillation analysis was performed at 25 °C to observe the elastic modulus and viscous modulus. Frequency (ω) was varied from 0.1 Hz to 10 Hz. All measurements were performed in the linear response region.

Solution apparent viscosity was measured by varying the shear rate (s^−1^) from 0.01 to 100 using a cone–plate geometry system at 25 °C. The same test conditions were used, and the concentration of all polymer solutions was 1750 mg/L.

### 3.4. Core Flooding Experiment

Core flooding experiments were carried out to study the mobility control and EOR ability of the polymer solutions at 45 °C.

The brine used in the experiment was a 4500 mg·L^−1^ NaCl solution. The permeability (*K*) of the porous media was calculated using Darcy’s law. The effective shear rate in porous media (${\dot{\gamma }}_{w}$) was estimated using Equation (7):

(7) $${\dot{\gamma }}_{w}=2.5\frac{4\nu }{r_{p}}$$

where *ν* is the interstitial mean velocity at the flow rate *Q* (*ν* = *Q*/(*S*·φ)), *S* is the cross-section of the core, *r*_p_ is the hydrodynamic capillary of the pore throat radius determined according to a corrected capillary model by permeability and porosity (φ) (i.e., by $r_{p}=1.15\cdot \sqrt{8k/\varphi }.$), and 2.5 is the geometric factor [19]. Given that the flow parameter is a function of the shear rate, the effective shear rate was set constant at 7.34 s^−1^ for all displacement processes. Artificial sandstone cores were used in the experiment, and the basic parameters are listed in Table 2.

The resistance factor (RF) and residual resistance factor (RRF) were used to evaluate the mobility control capability of the polymer solutions in porous media. The RF and RRF were calculated using Equations (8) and (9), respectively [12]

(8) $$RF=\frac{K_{w}/\mu_{w}}{K_{p}/\mu_{p}}$$

(9) $$RRF=\frac{K_{wb}}{K_{wa}}$$

where *K*_w_ is the brine permeability, mD; *K*_p_ is the polymer phase permeability, mD; *μ*_w_ is the aqueous viscosity, mPa·s; *μ*_p_ is the polymer phase viscosity, mPa·s; *K*_wb_ is the brine permeability before polymer flooding, mD; and *K*_wa_ is the brine permeability after polymer flooding.

Core flooding experiments were performed according to the following steps to study the EOR capability of the polymer solutions. First, the dried core was saturated with brine, and then crude oil was injected into the core until no water flowed out from the outlet. Second, water injection was performed until the water cut of the effluent reached 98%. Finally, a 0.3 pore volume (PV) polymer slug was injected into the core, followed by sequent water flooding until the water cut of the Finally, a 0.3 pore volume (PV) polymer slug was injected into the core, followed by sequential water flooding until the water cut of effluent reached 98% again effluent reached 98% again. The increased recovery by polymer flooding was calculated using Equation (10) [13]:

(10) $$EOR=E_{t}-E_{w}$$

where *E*_t_ is the total recovery of the entire oil displacement process (%), and *E*_w_ is the water injection recovery before polymer flooding (%).

## 4. Conclusions

The current study evaluated the potential of a host–guest inclusion system (β-CDP/HAP) in EOR compared with conventional HPAM and HAP solutions. Rheological experiments have proven that the polymer chains can form intermolecular associations through the inclusion of cyclodextrin and hydrophobes in the β-CDP/HAP solution, resulting in enhanced rheological properties. Specifically, the β-CDP/HAP solution exhibited variable activation energy and temperature-insensitive characteristics. This behavior may be related to the multiple supramolecular interactions within its network structure. As the temperature changed, the proportion of CD–hydrophobe inclusion structures and hydrophobic structures in the β-CDP/HAP solution also changed. This characteristic was not observed in the other CDP and HAP solutions. It may be attributed to the high content of β-CDP we used, which provided more cyclodextrin–hydrophobe binding sites. Therefore, it was easier to observe the changes in multiple network structures under varying temperatures. Given the noncovalent interaction, the β-CDP/HAP solution exhibited significant shear thinning behavior. Compared with the reference polymers, β-CDP/HAP exhibited a stronger mobility control ability and microscopic sweeping efficiency in porous media because of the enhanced intermolecular interaction and multilayer adsorption. The additional host–guest inclusion improved the EOR performance for the HAP solution. This work only evaluated the performance of the optimal host–guest inclusion system under the given conditions. The formulation can be adjusted according to reservoir conditions.

## Figures and Tables

**Table 1:** Parameters of the Carreau model for HPAM, HAP, and β-CDP/HAP.

Sample	*η*_0_ (mPa·s)	*λ*_S_ (s)	*γ*_c_ (s^−1^)	*n*
HPAM	47.5	0.52	1.94	0.77
HAP	624.5	0.64	1.57	0.40
β-CDP/HAP	1513.9	0.81	1.24	0.35

**Table 2:** Basic parameters of cores.

Core No.	Core Parameters				
	Diameter	Length	Porosity (φ)	Permeability	Initial Oil Saturation
	(cm)	(cm)	(%)	(mD)	(%)
1	3.804	7.69	18.93	884	-
2	3.801	7.71	19.16	917	-
3	3.810	7.68	19.42	846	-
4	2.50	30.16	20.91	1338	75.2
5	2.50	30.16	20.09	1515	76.3
6	2.50	30.16	20.84	1427	73.9

## Data Availability

The data that support the findings of this study are available.

## Supplementary Materials

The following supporting information can be downloaded at: https://www.mdpi.com/article/10.3390/molecules30010109/s1, Figure S1: NMR spectrum of β-CDP. Figure S2: Intrinsic viscosity and apparent viscosity of β-CDP with reaction time. Figure S3: TG data on β-CDP heated in air. Figure S4: TG data on β-cyclodextrin heated in air.

## References

[1] Afolabi R., Oluyemi G., Officer S., Ugwu J. (2019). Hydrophobically associating polymers for enhanced oil recovery–Part A: A review on the effects of some key reservoir conditions. J. Petrol. Sci. Eng..

[2] Taylor K., Nasr-El-Din H. Hydrophobically Associating Polymers for Oil Field Applications. Proceedings of the Canadian International Petroleum Conference.

[3] Habib S.H., Yunus R., Zakaria R., Biak D.R.A., Jan B.H.M., Amir Z. (2024). Chemical enhanced oil recovery: Synergetic mechanism of alkali, surfactant and polymer with overview of methyl ester sulfonate as a green alternative for EOR surfactant. Fuel.

[4] Li X., Pu X., Wei H. (2023). Enhanced oil recovery in fractured low-permeability reservoirs by a novel eel system prepared by sustained-release crosslinker and water-soluble thixotropic polymer. Geoenergy Sci. Eng..

[5] Zhu Y. Current developments and remaining challenges of chemical flooding EOR techniques in China. Proceedings of the SPE Asia Pacific Enhanced Oil Recovery Conference.

[6] Delamaide E., Tabary R., Rousseau D. Chemical EOR in low permeability reservoirs. Proceedings of the SPE EOR Conference at Oil and Gas West Asia.

[7] Lai N., Guo X., Zhou N., Xu Q. (2016). Shear resistance properties of modified nano-SiO2/AA/AM copolymer oil displacement agent. Energies.

[8] Lai N., Zhang Y., Zeng F., Wu T., Zhou N., Xu Q., Ye Z. (2016). Effect of Degree of Branching on the Mechanism of Hyperbranched Polymer to Establish the Residual Resistance Factor in High-Permeability Porous Media. Energy Fuels.

[9] Wever D., Picchioni F., Broekhuis A. (2011). Polymers for enhanced oil recovery: A paradigm for structure-property relationship in aqueous solution. Prog. Polym. Sci..

[10] Feng Y., Billon L., Grassl B., Khoukh A., François J. (2002). Hydrophobically associating polyacrylamides and their partially hydrolyzed derivatives prepared by post-modification 1. Synthesis and characterization. Polymer.

[11] Tang W., Zou C., Da C., Cao Y., Peng H. (2020). A review on the recent development of cyclodextrin-based materials used in oilfield applications. Carbohyd. Polym..

[12] Pu W., Liu R., Wang K., Li K., Yan Z., Li B., Zhao L. (2015). Water-soluble core-shell hyperbranched polymers for enhanced oil recovery. Ind. Eng. Chem. Res..

[13] Pu W., Yang Y., Wei B., Yuan C. (2016). Potential of a β-Cyclodextrin/Adamantane Modified Copolymer in Enhancing Oil Recovery through Host-Guest Interactions. Ind. Eng. Chem. Res..

[14] Wei B., Lu l., Li H., Xue Y. (2016). Novel wax-crystal modifier based on β-cyclodextrin: Synthesis, characterization and behavior in a highly waxy oil. J. Ind. Eng. Chem..

[15] Weickenmeier M., Wenz G., Huff J. (1997). Association thickener by host-guest interaction of a β-cyclodextrin polymer and a polymer with hydrophobic side-groups. Macromol. Rapid Comm..

[16] Li L., Guo X., Wang J., Liu P., Prud’Homme R., May B., Lincoln S. (2008). Polymer networks assembled by host-guest inclusion between adamantyl and β-cyclodextrin substituents on poly (acrylic acid) in aqueous solution. Macromolecules.

[17] Crini G. (2021). Cyclodextrin–epichlorohydrin polymers synthesis, characterization and applications to wastewater treatment: A review. Environ. Chem. Lett..

[18] Wilson L.D., Guo R. (2012). Preparation and sorption studies of polyester microsphere copolymers containing β-cyclodextrin. J. Colloid Interface Sci..

[19] Chauveteau G. (1982). Rodlike Polymer Solution Flow through Fine Pores: Influence of Pore Size on Rheological Behavior. J. Rheol..

[20] Sun F., Huang Q., Wu J. (2014). Rheological behaviors of an exopolysaccharide from fermentation medium of a Cordyceps sinensis fungus (Cs-HK1). Carbohyd. Polym..

[21] Ait-Kadi A., Carreau P., Chauveteau G. (1987). Rheological Properties of Partially Hydrolyzed Polyacrylamide Solutions. J. Rheol..

[22] Zhang Y., Feng Y., Li B., Han P. (2019). Enhancing oil recovery from low-permeability reservoirs with a self-adaptive polymer: A proof-of-concept study. Fuel.

[23] Regalado E., Selb J., Candau F. (1999). Viscoelastic behavior of semidilute solutions of multisticker polymer chains. Environ. Macromol..

[24] Candau F., Selb J. (1999). Hydrophobically-modified polyacrylamides prepared by micellar polymerization. Adv. Colloid. Interfac..

[25] Volpert E., Selb J., Candau F. (1998). Associating behaviour of polyacrylamides hydrophobically modified with dihexylacrylamide. Polymer.

[26] Kujawa P., Audibert-Hayet A., Selb J., Candau F. (2004). Rheological properties of multisticker associative polyelectrolytes in semidilute aqueous solutions. J. Polym. Sci. Pol. Phys..

[27] Peng J., Dong R., Ren B., Chang X., Tong Z. (2014). Novel hydrophobically modified ethoxylated urethanes end-capped by percec-type alkyl substituted benzyl alcohol dendrons: Synthesis, characterization, and rheological behavior. Macromolecules.

[28] Beheshti N., Kjøniksen A., Zhu K., Knudsen K., Nyström B. (2010). Viscosification in polymer-surfactant mixtures at low temperatures. J. Phys. Chem. B.

[29] Wu D., Cheng J., Su X., Feng Y. (2021). Hydrophilic modification of methylcellulose to obtain thermoviscosifying polymers without macro-phase separation. Carbohyd. Polym..

[30] Liu X., Jiang W., Gou S., Ye Z., Feng M., Lai N., Liang L. (2013). Synthesis and evaluation of novel water-soluble copolymers based on acrylamide and modular β-cyclodextrin. Carbohyd. Polym..

[31] Wei B. (2015). β-Cyclodextrin associated polymeric systems: Rheology, flow behavior in porous media and enhanced heavy oil recovery performance. Carbohyd. Polym..

[32] Gogarty W. (1967). Mobility Control with Polymer Solutions. Soc. Petrol. Eng. J..

[33] Minko S., Müller M., Motornov M., Nitschke M., Grundke K., Stamm M. (2003). Two-level structured self-adaptive surfaces with reversibly tunable properties. J. Am. Chem. Soc..

[34] Dupuis G., Rousseau D., Tabary R., Grassi B. (2011). Flow of hydrophobically modified water-soluble-polymer solutions in porous media: New experimental insights in the diluted regime. SPE J..

[35] Dupuis G., Rousseau D., Tabary R., Grassl B. Injectivity of hydrophobically modified water soluble polymers for IOR: Controlled resistance factors vs. flow-induced gelation. Proceedings of the SPE International Symposium on Oilfield Chemistry.

[36] Reichenbach-Klinke R., Stavland A., Langlotz B., Wenzke B., Brodt G. New insights into the mechanism of mobility reduction by associative type copolymers. Proceedings of the SPE Enhanced Oil Recovery Conference.

[37] Renard E., Barnathan G., Deratani A., Sebille B. Characterization and Structure of Cyclodextrin -Epichlorohydrin Polymers—Effects of Synthesis Parameters. Proceedings of the Eighth International Symposium on Cyclodextrins.

[38] Renard E., Deratani A., Volet G., Sebille B. (1997). Preparation and characterization of water soluble high molecular weight β-cyclodextrin-epichlorohydrin polymers. Eur. Polym. J..

