# Supplementary material for: Performance Evaluation of Enhanced Oil Recovery by Host–Guest Interaction of β-Cyclodextrin Polymer/Hydrophobically Associative Polymer
Source: Molecules. 2024 Dec 30;30(1):109. doi: 10.3390/molecules30010109 (PMC11721016; doi:10.3390/molecules30010109)
Supplement: Supplementary file 1 [file molecules-30-00109-s001.zip › molecules-3305168-supplementary.pdf]

Supporting Information for

Performance evaluation of enhanced oil recovery by host-guest interaction of β-cyclodextrin polymer/hydrophobically associative polymer

Xi Li[1,2,*], Zhongbing Ye[1,2], Pingya Luo[1]

1. State Key Laboratory of Oil and Gas Reservoir Geology and Exploitation, Southwest Petroleum University, Chengdu, Sichuan 610500, PR China

2. School of Materials and Environmental Engineering, Chengdu Technological University, Chengdu, Sichuan 610031, PR China

* Correspondence: Email: lixifantasy@sina.com (X.L.)

1. NMR spectrum of β-CDP

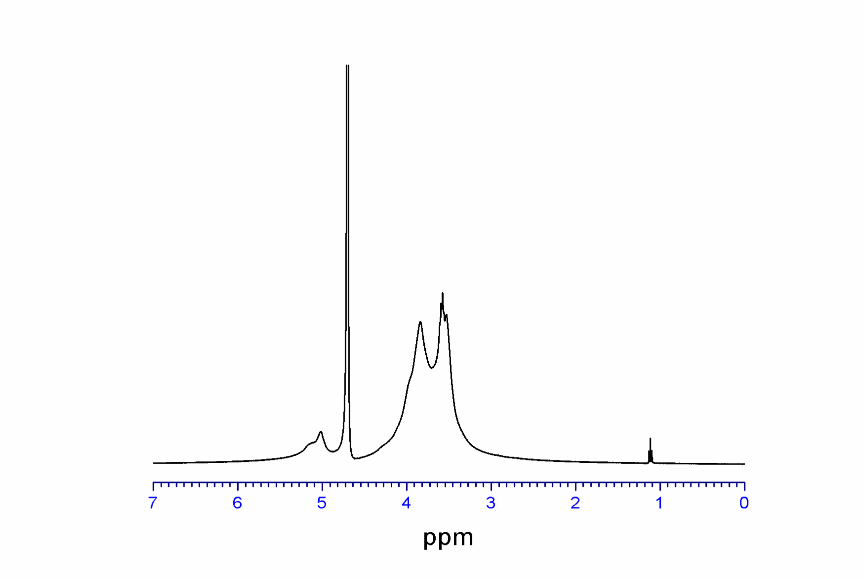

Figure S1. NMR spectrum of β-CDP

2. Intrinsic viscosity and apparent viscosity of β-CDP with reaction time

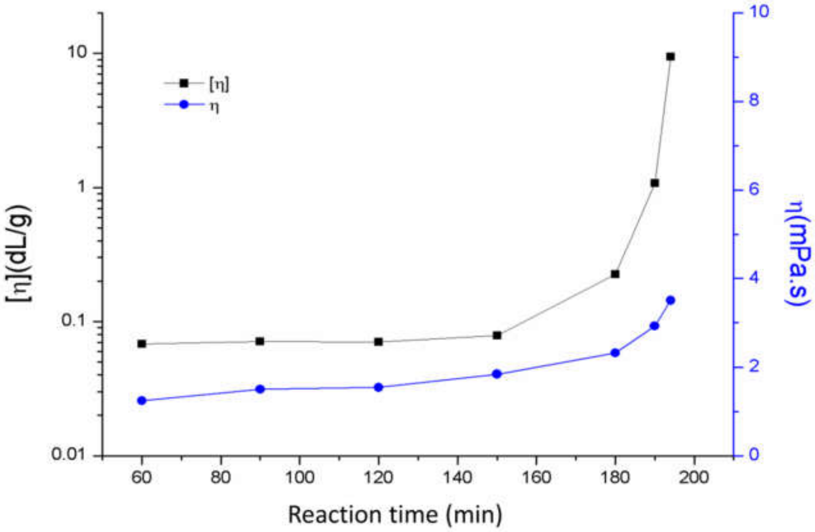

Figure S2. Intrinsic viscosity and apparent viscosity of β-CDP with reaction time

In the first 120 min of the reaction, epichlorohydrin modified the hydroxyl group of cyclodextrin to form tail chains with different lengths and the intrinsic viscosity (molecular weight) of the product changed smoothly. As the reaction progressed, the intrinsic viscosity continued to rise, indicating that the polycondensation starts giving low Mw compounds. Finally, the low Mw compounds condense together leading to high Mw polymers. The intrinsic viscosity increased sharply 15 min before gelation.

The products of β-CDP with different termination times were obtained and formulated into solutions of 2000mg/L. The apparent viscosity of the solutions was measured, and the viscosity of the solution gradually increased with reaction time. However, the viscosity of β-CDP solutions obtained for each reaction time was relatively small, which indicates that the hydrodynamic radius is small and the conformation of β-CDP molecules is quite tight.

3. TG data on β-CDP heated in air

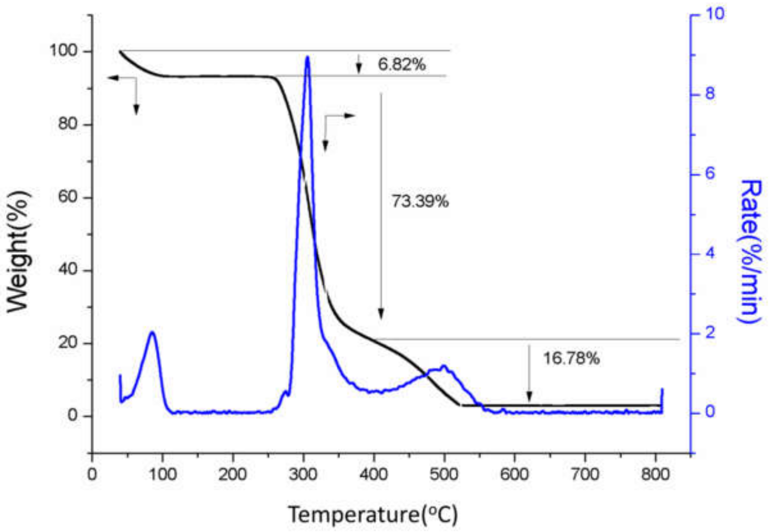

Figure S3. TG data on β-CDP heated in air

The thermogravimetric loss process of β-CDP can be divided into three stages: the first stage (the temperature below 110 °C), the thermogravimetric loss is mainly due to the loss of crystallization water and adsorption water; the second stage (the temperature ranges from 250 to 390 °C), where the thermogravimetric loss is reduced by 73.39%. Generally, the introduction of modification groups to cyclodextrins is helpful for the thermal stability of cyclodextrins. In the third stage (the temperature above 400 °C), the thermal oxidation process of the residue is relatively slow. The sample weight does not change when the temperature exceeds 530 °C.

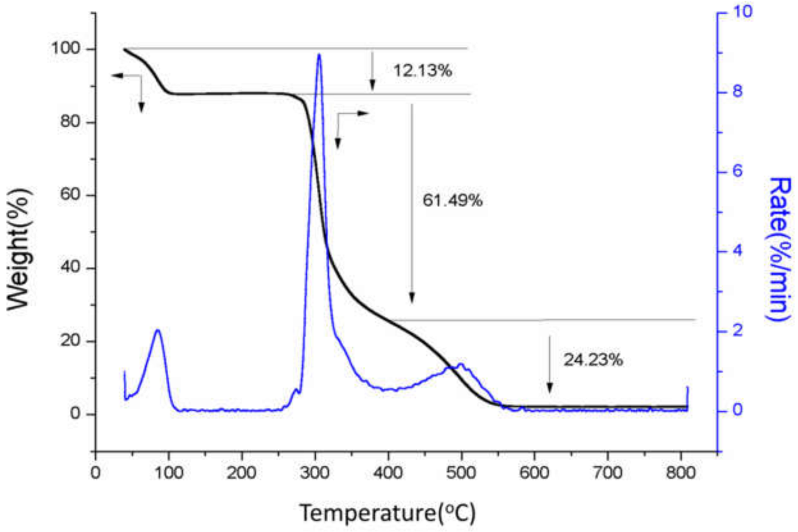

Figure S4. TG data on β-cyclodextrin heated in air
